# Epigenetic regulation of histone modifications and *Gata6* gene expression induced by maternal diet in mouse embryoid bodies in a model of developmental programming

**DOI:** 10.1186/s12861-015-0053-1

**Published:** 2015-01-21

**Authors:** Congshan Sun, Oleg Denisenko, Bhavwanti Sheth, Andy Cox, Emma S Lucas, Neil R Smyth, Tom P Fleming

**Affiliations:** Centre for Biological Sciences, University of Southampton, Mailpoint 840, Level D Lab & Path Block, Southampton General Hospital, Tremona Road, Southampton, SO16 6YD UK; Department of Medicine, University of Washington, Seattle, WA 98109 USA

**Keywords:** Maternal low protein diet, Embryoid body, Mouse blastocyst, Histone epigenetics, Metabolic disease, Gata6, Primitive endoderm, Chromatin immunoprecipitation

## Abstract

**Background:**

Dietary interventions during pregnancy alter offspring fitness. We have shown mouse maternal low protein diet fed exclusively for the preimplantation period (Emb-LPD) before return to normal protein diet (NPD) for the rest of gestation, is sufficient to cause adult offspring cardiovascular and metabolic disease. Moreover, Emb-LPD blastocysts sense altered nutrition within the uterus and activate compensatory cellular responses including stimulated endocytosis within extra-embryonic trophectoderm and primitive endoderm (PE) lineages to protect fetal growth rate. However, these responses associate with later disease. Here, we investigate epigenetic mechanisms underlying nutritional programming of PE that may contribute to its altered phenotype, stabilised during subsequent development. We use embryonic stem (ES) cell lines established previously from Emb-LPD and NPD blastocysts that were differentiated into embryoid bodies (EBs) with outer PE-like layer.

**Results:**

Emb-LPD EBs grow to a larger size than NPD EBs and express reduced *Gata6* transcription factor (regulator of PE differentiation) at mRNA and protein levels, similar to Emb-LPD PE derivative visceral yolk sac tissue *in vivo* in later gestation. We analysed histone modifications at the *Gata6* promoter in Emb-LPD EBs using chromatin immunoprecipitation assay. We found significant reduction in histone H3 and H4 acetylation and RNA polymerase II binding compared with NPD EBs, all markers of reduced transcription. Other histone modifications, H3K4Me2, H3K9Me3 and H3K27Me3, were unaltered. A similar but generally non-significant histone modification pattern was found on the *Gata4* promoter. Consistent with these changes, histone deacetylase *Hdac-1,* but not *Hdac-3,* gene expression was upregulated in Emb-LPD EBs.

**Conclusions:**

First, these data demonstrate ES cells and EBs retain and propagate nutritional programming adaptations *in vitro*, suitable for molecular analysis of mechanisms, reducing animal use. Second, they reveal maternal diet induces persistent changes in histone modifications to regulate *Gata6* expression and PE growth and differentiation that may affect lifetime health.

## Background

Periconceptional environment, especially during oocyte maturation and preimplantation development, can influence the pattern of later gestation leading to permanent changes in offspring growth, physiology, health and disease risk through to adulthood [1-3]. Factors such as the quality and composition of maternal or paternal diet, parental metabolism and health, or specific conditions as used in assisted conception such as embryo culture, can all influence the developmental programme. This sensitive window in the lifecycle around conception can be viewed within the broader context of the Developmental Origins of Health and Disease (DOHaD) concept. This proposes that risk of adult onset diseases may derive from *in utero* conditions where nutrient availability may control fetal growth and metabolic homeostasis but which may predispose to adult disease, particularly cardiovascular dysfunction and metabolic syndrome, if homeostatic changes do not match postnatal environment. Epidemiological studies on human populations and various animal models show support for the DOHaD concept [4-7].

We have used a rodent maternal low protein diet model to study mechanisms of periconceptional programming whereby protein restriction is applied exclusively during the period from mating to blastocyst formation (Emb-LPD, 9% casein, E0-3.5 in mouse) with normal nutrition (NPD, 18% casein) provided for the remainder of gestation, and standard chow diet postnatally. This brief nutritional challenge is sufficient to induce cardiometabolic dysfunction, hypertension and abnormal behaviour in adulthood [8,9]. Emb-LPD changes the pattern of development by altering the composition of the uterine fluid which is detected by blastocysts via mTOR signalling [10]. The embryo responds to the nutrient challenge by activating several compensatory processes within extra-embryonic lineages which collectively act to increase nutrient provision from the mother for the remainder of gestation to protect fetal growth. These responses include increased endocytosis and proliferation within the trophectoderm lineage (TE; progenitor of chorio-allantoic placenta) and increased motility and invasiveness of outgrowing trophoblast at the time of implantation [10,11]. LPD treatment maintained throughout gestation leads to increased nutrient transport efficiency in the mid- and late-gestation placenta [12]. Stimulated endocytosis is also seen in response to Emb-LPD in the primitive endoderm (PE) extra-embryonic lineage formed from the blastocyst inner cell mass (ICM); this response is maintained until late gestation within the derivative visceral endoderm of the yolk sac placenta to promote nutrient uptake from the uterine milieu [9,11]. Nutrient provision and growth promotion resulting from these extra-embryonic adaptations to poor maternal diet, whilst likely favouring competitive fitness of offspring during periods of limited food supply, also lead to later chronic disease when the diet improves, evidenced by the resulting perinatal weight correlating with adult CV and behavioural dysfunction [9].

Since extra-embryonic responses to Emb-LPD persist from early development throughout gestation and have important consequences for protecting conceptus growth and affecting adult disease risk, we anticipate conserved epigenetic mechanisms may be driving these physiological processes. Moreover, the compensatory changes persist well beyond the period of dietary challenge and reflect a ‘memory’ of an earlier environment. Periconceptional induction of epigenetic change has been demonstrated in other models of programming, such as following *in vitro* culture treatment of pre-implantation embryos [13-17]. However, clear evidence of epigenetic modifications driving physiological responses within an *in vivo* periconceptional programming model has not been forthcoming previously.

Here, we investigate the epigenetic status of histone modifications occurring within the PE lineage in response to Emb-LPD for evidence of the programming of altered phenotype. We have used embryoid bodies (EBs) derived from embryonic stem (ES) cell lines generated from Emb-LPD and NPD blastocysts since the PE-like layer on the surface of Emb-LPD EBs exhibit the enhanced endocytosis compensatory phenotype after at least six passages in standard culture [11].

## Results

### Effect of maternal diet on EB size

**Figure 1:**
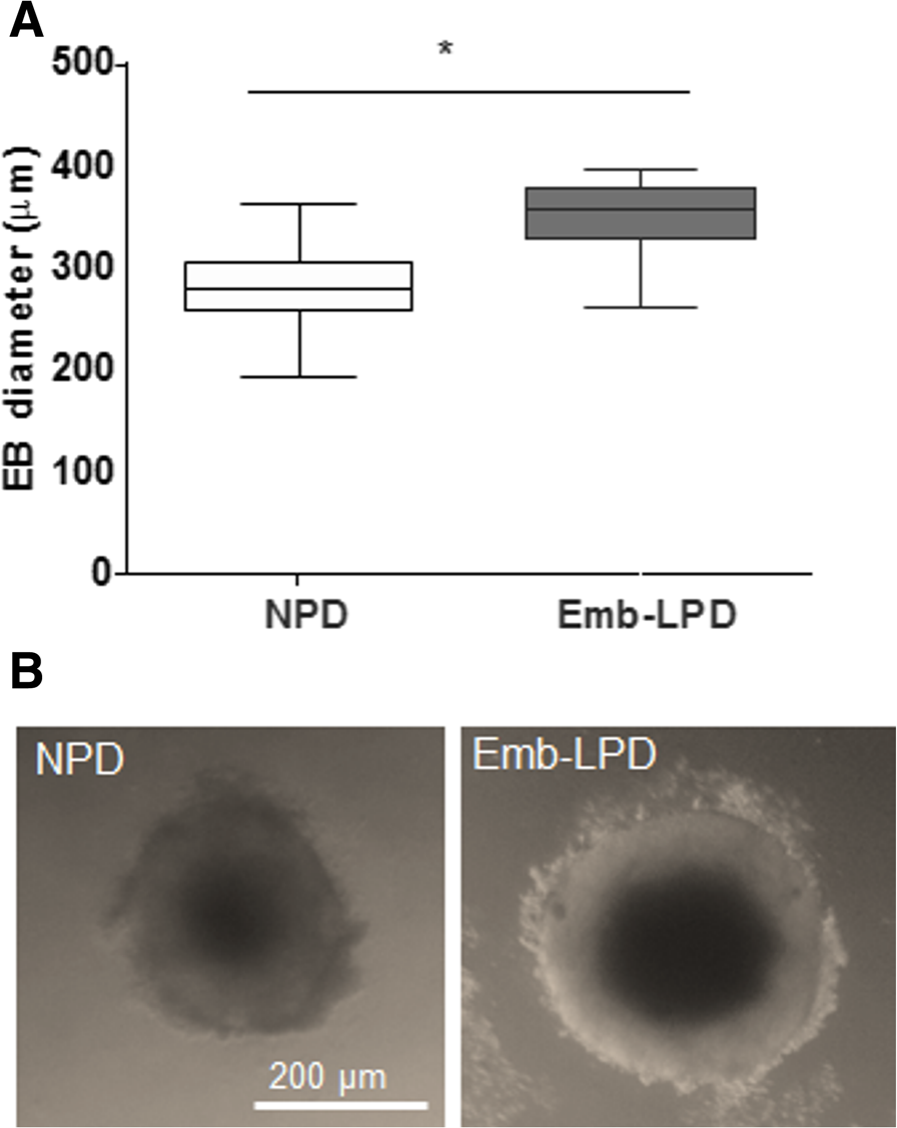
**Embryoid bodies formed from ES cell lines derived from Emb-LPD blastocysts grow to a larger size that those from NPD blastocysts. (A)** Embryoid body size at day 5.5 culture measured by diameter, presented as mean ± upper and lower quartiles (box) and SEM (vertical lines). Data from 6 cell lines as biological replicates per treatment with 5 embryoid bodies measured per replicate (ie, 30 per treatment); each rounded EB was measured twice at orthogonal positions and the mean recorded. **(B)** Representative images of NPD and Emb-LPD EBs. *p < 0.05.

ES cells derived from blastocysts collected from Emb-LPD and NPD females were maintained from passage 6 for 5.5 days in culture for EB formation in 96-well low adhesion plate culture. At this time point, EBs have formed primitive endoderm-like (PE) layer on their surface as demonstrated by the presence of Gata6 and Dab2 marker proteins [11]. EBs were imaged and diameters measured. EBs differentiated from Emb-LPD ES cells were significantly larger (~15%; p < 0.05) than NPD EBs (Figure 1).

### Effect of maternal diet on embryoid body Gata factor expression

**Figure 2:**
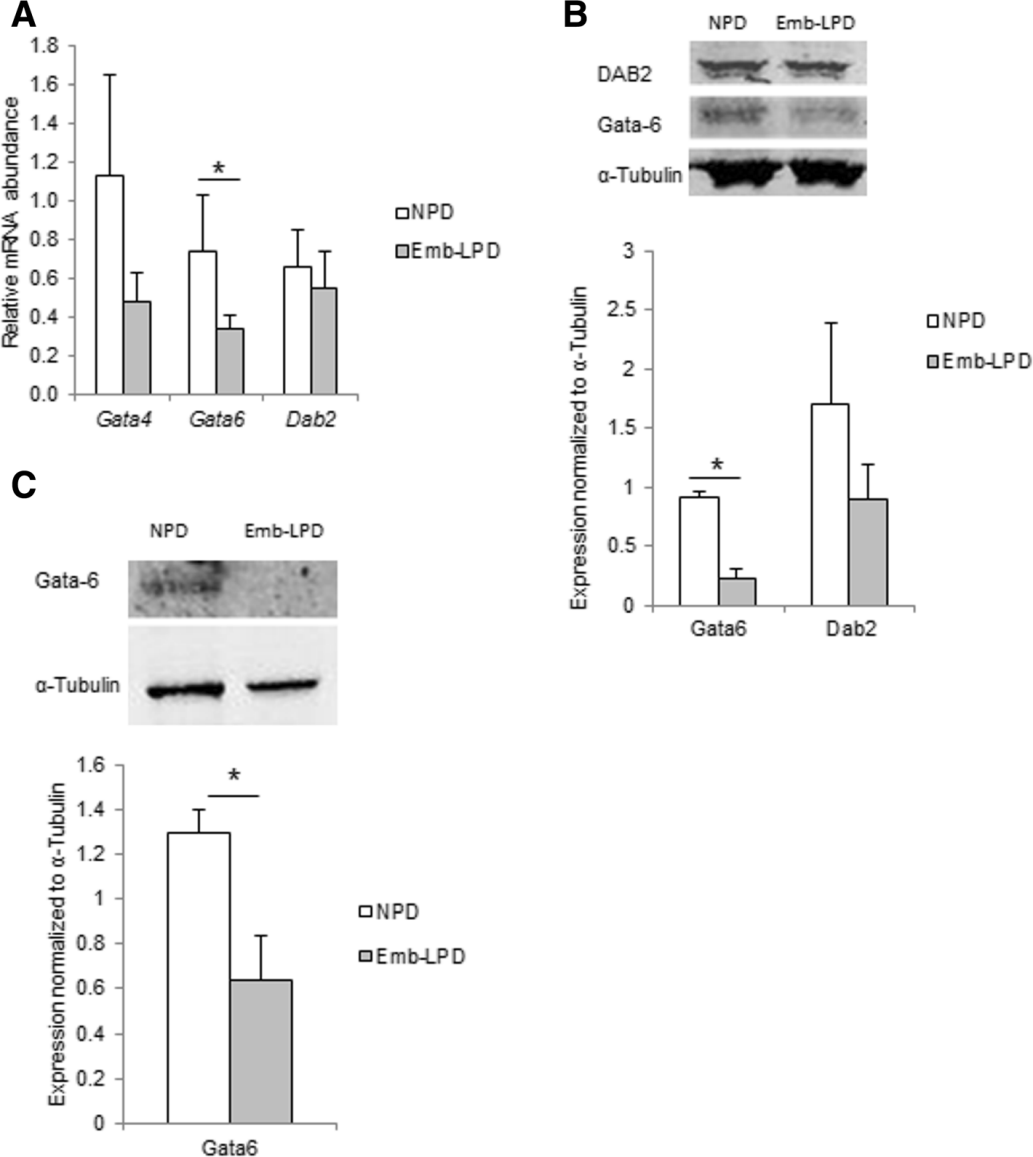
**Gata factor expression in embryoid bodies (EBs) at day 5.5 culture and in ex vivo visceral yolk sac (VYS) at E17.5 in relation to maternal diet. (A)** Expression of *Gata4*, *Gata6* and *Dab2* mRNA in EBs of Emb-LPD and NPD groups presented as ratio to the geometric mean of *Gapdh* and *Ppib* transcripts (n = 5 per treatment). **(B)** Expression of Gata6 and Dab2 protein in EBs from NPD and Emb-LPD groups (n = 6 cell lines per treatment). Upper: representative images of protein immunoblot bands. Lower: band intensity normalized to α-tubulin expression. **(C)** Expression of Gata6 protein in VYS from Emb-LPD, LPD and NPD group (n = 4 samples per treatment). Upper: representative images of protein immunoblot bands. Lower: band intensity normalized to α-tubulin expression. Values presented are mean ± SEM. *p < 0.05.

The PE lineage is regulated through *Gata6* and *Gata4* transcription factors that activate PE specification and differentiation through several downstream target genes [18-21]. We investigated the expression of *Gata4* and *Gata6* and the downstream target gene, *Dab2* in Emb-LPD and NPD EBs. *Gata6* gene expression was significantly reduced in Emb-LPD EBs while *Gata4* was reduced but only to trend level and *Dab2* expression was unaffected by maternal dietary origin (Figure 2A). Reduced protein expression of Gata6 but not Dab2 was also evident in Emb-LPD EBs (Figure 2B). The authenticity of the changed *Gata6* expression in Emb-LPD EBs was supported by *ex vivo* analysis of mouse E17 visceral yolk sac tissue also showing reduced Gata6 protein expression compared with NPD control (Figure 2C).

### Effect of maternal diet on *Gata6* promoter histone acetylation in embryoid bodies

**Figure 3:**
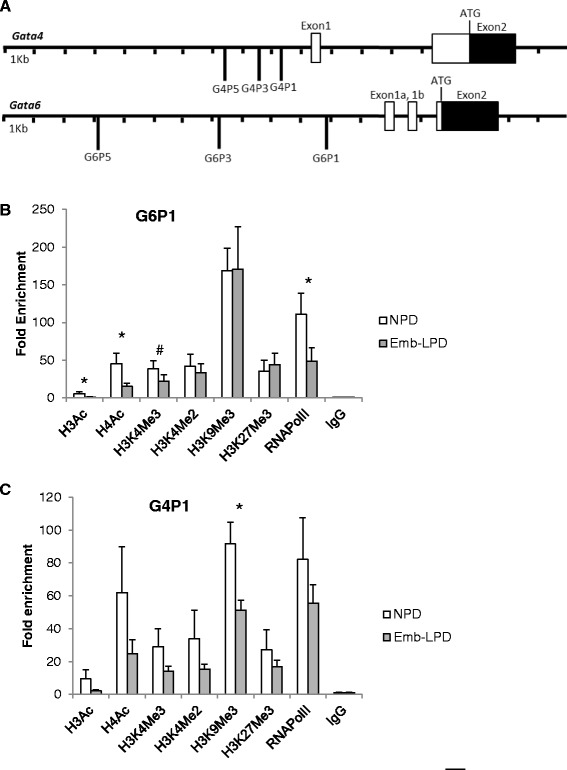
**ChIP analysis of histone modifications at Gata factor G6P1 and G4P1 promoter loci in EBs in relation to maternal diet. (A)** The *Gata4* gene 5’ region is illustrated with three regions 5’ of exon 1 amplified by G4P1, G4P3 and G4P5. The *Gata6* gene 5’ region is illustrated with three regions amplified by G6P1, G6P3 and G6P5. **(B, C)** ChIP analysis was performed using antibodies to H3Ac, H4Ac, H3K4Me3, H3K4Me2, H3K9Me3 H3K27Me3 and RNA Polymerase II and quantified by real-time PCR amplifying the G6P1 **(B)** and G4P1 **(C)** loci with results presented as fold enrichment over IgG. Duplicates of ChIP experiments were performed throughout for verification. Values are means from 6 cell lines from each diet group with standard errors represented by vertical bars, *p < 0.05, # < 0.1.

**Figure 4:**
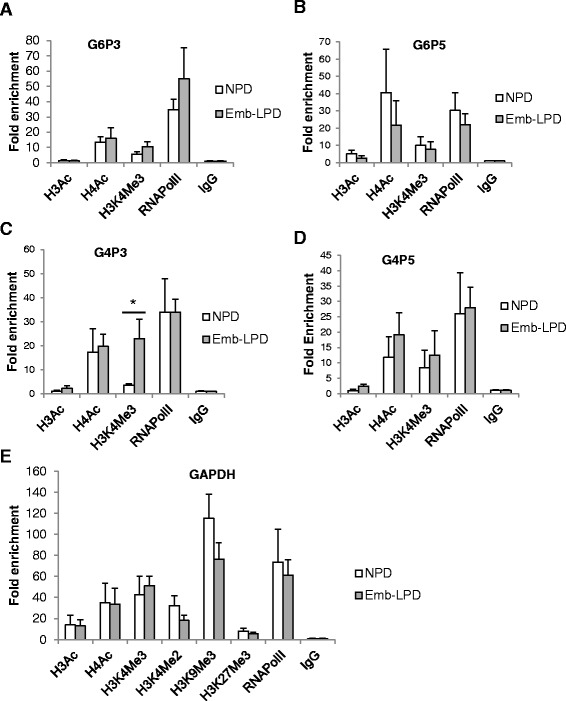
**ChIP analysis of histone modifications at Gata factor G6P3, G6P5, G4P3 and G4P5 promoter loci in EBs in relation to maternal diet. (A-D)** ChIP analysis was performed using antibodies H3Ac, H4Ac, H3K4Me3 and RNA polymerase II and quantified by real-time PCR amplifying the G6P3 **(A)**, G6P5 **(B)**, G4P3 **(C)** and G4P5 **(D)** loci with results presented as fold enrichment. **(E)** ChIP analysis was performed using antibodies H3Ac, H4Ac, H3K4Me3, H3K4Me2, H3K9Me3 H3K27Me3 and RNA Polymerase II and quantified by real-time PCR amplifying the *Gapdh* promoter with results presented as fold enrichment over IgG. Duplicates of ChIP experiments were performed throughout for verification. Values are means for 6 cell lines from each diet group with standard errors represented by vertical bars, *p < 0.05, # < 0.1.

Histone modifications have been shown to regulate *Gata4* and *Gata6* gene expression in other models [22,23]. We used ChIP assay to examine three upstream regions of the *Gata4* and *Gata6* genes (Figure 3A) in EBs to compare levels of histone modifications within promoter domains with respect to maternal diet. A panel of antibodies was used to probe histone acetylation and methylation with targets for acetylated H3 and H4, H3K4Me3, H3K4Me2, H3K9Me3 and H3K27Me3. Our analysis revealed a distinct pattern of modifications dependent upon dietary origin of EBs. The *Gata6* promoter at G6P1 site in Emb-LPD EBs exhibited significant hypoacetylation of both H3 and H4 and a trend of decrease in the density of the histone marker H3K4Me3 compared with NPD EBs (Figure 3B). In addition to these three histone modification changes known to be associated with reduced gene expression [24], the G6P1 *Gata6* promoter site in Emb-LPD EBs had reduced enrichment of RNA polymerase II (Figure 3B), further supporting a suppressed state of expression. No significant changes in other histone modifications, including H3K4Me2, H3K9Me3 and H3K27Me3, were detected. The *Gata4* promoter site at G4P1 showed a pattern of histone modifications in Emb-LPD EBs similar to those in the *Gata6* promoter but not to the level of significance except that H3K9Me3 was significantly reduced compared with NPD EBs (Figure 3C). At other sites upstream of the *Gata6* and *Gata4* promoter domains (G6P3, G6P5, G4P3, G4P5), maternal diet had little or no effects on histone modifications (Figure 4A-D); however, H3K4Me3 on G4P3 showed a significant increase in Emb-LPD EBs (Figure 4C). We used *Gapdh* as a control house-keeping gene in our ChIP analysis and found no difference in histone modifications on its promoter in EBs with respect to maternal diet (Figure 4E).

### Effect of maternal diet on expression of histone deacetylases in embryoid bodies

**Figure 5:**
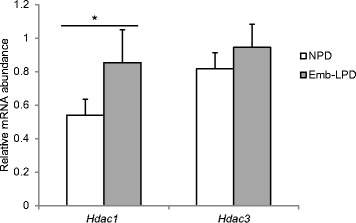
**Gene expression of histone modification enzymic regulators** ***Hdac-1*** **and** ***Hdac-3*** **in embryoid bodies (EBs) at day 5.5 culture in relation to maternal diet.** Expression presented as ratio to the geometric mean of *Gapdh* and *Ppib* transcripts. (n = 5 per treatment). *p < 0.05.

Given the pattern of histone hypoacetylation detected at the *Gata6* promoter coinciding with reduced expression of this gene in Emb-LPD EBs, we evaluated whether the expression of histone deacetylases (HDACs) was altered in response to maternal diet. HDACs are expressed in early embryos and have been shown to modify their expression in response to *in vitro* culture [25]. We found *Hdac-1* gene expression but not *Hdac-3* was upregulated in Emb-LPD EBs (Figure 5).

## Discussion

In this study we have investigated the molecular mechanisms of developmental programming of the PE extra-embryonic lineage within our Emb-LPD mouse model associated with adult-onset disease. The Emb-LPD PE shows enhanced endocytosis at ligand, lysosomal and receptor levels, a cellular modification that is sustained through to late gestation in the yolk sac visceral endoderm to support increased nutrient uptake despite poor maternal nutrition, thereby likely to protect fetal development [9,11]. Significantly, these changes are maintained after induction even if maternal diet is returned to control levels, a characteristic we have demonstrated also occurs in the trophectoderm (TE) extra-embryonic lineage [10,11] and is suggestive of epigenetic mechanisms, the focus of the current study. We chose to investigate potential PE epigenetic mechanisms using EBs since they maintain the enhanced endocytosis cellular phenotype induced by nutritional programming previously revealed from *ex vivo* tissues [9,11] and, in the current study, show similar downregulation of *Gata6* as seen in *ex vivo* VYS, thereby confirming their authenticity. Also, the PE-like layer formed on EBs from undifferentiated ES cells is representative of the PE layer formed in the late blastocyst from the ICM, and PE-like cells in EBs as well as the core primitive ectoderm have been used extensively as a model for early cell lineage derivation in post-implantation development over many years [26,27].

Our results show significant change in gene expression and histone modifications in Emb-LPD EBs in comparison with NPD EBs. Emb-LPD EBs express reduced levels of *Gata6* transcription factor, a key regulator of PE differentiation [18,19], consistent with *Gata6* downregulation found *in vivo* within the Emb-LPD VYS. Reduced *Gata6* expression coincided with histone H3 and H4 hypoacetylation and reduced recruitment of RNA polymerase II at the *Gata6* promoter in Emb-LPD EBs, all factors consistent with reduced gene expression [24]. Moreover, the expression of *Hdac-1* but not *Hdac-3* was increased in Emb-LPD EBs. These HDACs are known to be expressed in early embryos and are sensitive to culture conditions so represent good candidates for coordinating histone epigenetic programming [25]. Collectively, these data reveal for the first time a persistent change in EB epigenetic status mediated through maternal diet and provide new clues to the origin of developmental programming mechanisms in this model. They also demonstrate that ES cells and derivative EBs maintain altered gene expression *in vitro*, long after the inductive dietary challenge, and provide a useful tool for analysis of underlying mechanisms, reducing the requirement for experimental animals.

The finding that Emb-LPD EBs, and *ex vivo* VYS, exhibit reduced *Gata6* expression, apparently regulated through histone hypoacetylation in the EBs, may explain the increased size of Emb-LPD EBs. *Gata6* and *Gata4* are zinc-finger transcription factors that perform multiple roles both during development in the determination of cell lineages and in adult tissues in maintaining cell differentiation states [28,29]. Loss of Gata factor expression has been implicated in several forms of cancer and in ovarian cancer models where loss of *Gata6* and *Gata4* expression coincides with *Gata6* and *Gata4* promoter histone hypoacetylation in response to HDAC activity coupled with growth promotion and malignancy [23,30,31] indicating a similarity in epigenetic and cellular mechanisms to the current study.

Whilst Emb-LPD EBs had downregulated *Gata6* expression, *Gata4* expression was not significantly affected. In ES cells, absence of *Gata6* gene leads to loss of *Gata4* expression whilst absence of *Gata4* gene does not inhibit *Gata6* expression, indicating a hierarchical relationship [21,32,33]. However, functional redundancy exists between *Gata6* and *Gata4* expression in other models including pancreatic and ovarian germ cell differentiation and in EBs during myocyte differentiation with *Gata4* expression not dependent upon *Gata6* expression [34-36]. Thus, the distinction between *Gata6* and *Gata4* expression in the current study may be explained either by functional redundancy or by *Gata6* expression, although reduced, being above the threshold required for *Gata4* expression.

The Emb-LPD EBs also showed expression of the *Gata6* downstream target gene, *Dab2*, required for epithelial function especially in receptor-mediated endocytosis [37]. Dab2 acts as a cargo-selective adaptor protein facilitating apical localisation of the megalin receptor (*Lrp2* gene) and clathrin-mediated endocytosis [37]. The endocytic function in Emb-LPD EBs is stimulated together with increased expression of megalin as a compensatory response to maternal diet [11] and *Dab2* expression and function is likely protected in the EB model to achieve this. *Dab2* expression may be maintained either by a *Gata4*-dependent pathway [33,38], by *Gata6* being above the threshold required for *Dab2* promoter activation or by an alternative mechanism. We anticipate that the growth stimulation coinciding with reduced expression of *Gata6* but not *Dab2* in Emb-LPD EBs will be partly driven by the increased nutrient delivery provided by enhanced endocytosis. The resulting increase in growth in Emb-LPD EBs may extend to both surface PE-like layer and core epiblast cells and, like other compensatory response mechanisms, may reflect *in vivo* processes that safeguard fetal development against dietary deficiency. Indeed, the increased endocytic activity observed within the mature VYS in late gestation may be dependent upon this initial growth stimulation.

## Conclusions

We have shown that maternal diet regulates the epigenetic status of the early embryo, reducing expression of the PE lineage regulator, *Gata6* transcription factor, in EBs. Reduced *Gata6* gene expression coincides with histone hypoacetylation and loss of RNA polymerase binding of the *Gata6* promoter, and stimulation of *Hdac-1* expression. These changes are associated with increased growth of the Emb-LPD EB which may contribute, alongside stimulation in endocytosis, as compensatory responses to support maintenance of nutrient delivery.

## Methods

### Ethics statement

All animal research was conducted under UK Home Office project license and local ethics approval (University of Southampton).

### Animals, diet treatment and embryo collection

MF1 mice were bred in-house (University of Southampton Biomedical Research Facility) on a 0700–1900 light cycle with standard chow, under UK Home Office license and local ethics approval. Virgin females (7–8.5 weeks) were mated naturally overnight with MF1 males and plug positive females were housed individually the following morning and assigned randomly to either normal protein diet (18% casein, NPD) or isocaloric low protein diet (9% casein, Emb-LPD) until embryonic day 3.5 (E3.5); diet composition has been described elsewhere [8,9]. Embryos were collected at the blastocyst stage after cervical dislocation and uterine flushing with H6 medium with 4 mg/ml BSA (H6 + BSA) [39].

### ES cell culture and embryoid body (EB) formation

Mouse embryonic stem (ES) cell lines were prepared using standard procedures from blastocysts derived from mothers fed NPD or Emb-LPD upon culture in knockout-Dulbecco’s modified Eagle medium [high glucose] (DMEM [high glucose], Gibco).

including 20% knock out serum replacement (Gibco) with other supplements and were subsequently maintained and expanded on mouse embryonic fibroblast feeder layers up to passage 5–7 as described in detail elsewhere [11]. A total of 18 ES lines were generated from Emb-LPD blastocysts and 38 lines from NPD blastocysts. Six clones, each from a different mother and of male gender and normal karyotype, from each diet treatment were selected for use in the current study and were used for all relevant experiments. For embryoid body (EB) formation, ES cells were dissociated with 0.05% trypsin-EDTA and suspended in ES cell culture medium without leukaemia inhibitory factor (LIF) supplementation for 1 h on gelatin-treated dishes. A cell suspension (4,000 in 200 μl) was subsequently pipetted into low-adherence 96-well plates and statically incubated at 37°C in humidified air with 5% CO_2_ for 5.5 days to form individual EBs within each well using a method previously described that is optimised for uniform EB size production [11,40]. EB diameter at specific time intervals was measured using an Olympus microscope software and Cell sense®.

### RNA isolation and real-time PCR

RNA isolation and quantitative real-time PCR (qRT-PCR) of EBs and tissues was performed as described previously [41]. Briefly, total RNA was extracted from EBs and tissues using the RNeasy Mini kit (Qiagen, UK), with on-column DNase digestion. RNA was quantified using the Nanodrop ND-1000 spectrophotometer, and cDNA generated using a random priming strategy and the ImProm-II™ Reverse Transcription System (Promega, UK). cDNA was diluted to a concentration equivalent to 5 ng RNA per μl and used at 1 μl in a reaction volume of 20 μl with forward and reverse primers at final concentration of 300 nM each in qRT-PCR using the Chromo4 Real-Time Detector (BioRad, UK) with Opticon Monitor v3.1 software. Thermal cycling conditions were 95°C 10 min enzyme activation, then 40 cycles of 95°C for 15 s followed by 60°C for 1 min, with a final extension step of 10 min at 72°C. Primers used for qRT-PCR were designed by Primer3 software (Table 1). For EBs, *Gapdh* and *Ppib* were selected from 6 house-keeping candidates with GeNorm and NormFinder software for stability, showing no change in expression between Emb-LPD and NPD treatments (Table 2). For quantification, efficiency of primers was determined by series 1/10 dilution and Ct value. Efficiency was calculated as E = 10^1/slope^ and qualified primer efficiency is between 1.9-2.1. Calculation of relative expression of target gene was calculated as E^^-dCt^ then divided by the geometric mean of relative expression of the reference gene pair.

**Table 1:** **Primers used in qRT-PCR and ChIP Q-PCR**

**Primer name**	**Forward primer (5′-3′)**	**Reverse perimer (5′-3′)**	**Assay**
*Gata4 P1*	gggctggtggaggttctc	tcagtgcctagagacgcaag	ChIP Q-PCR
*Gata4 P3*	gccattctctgcattcatcc	tcgctgagcatcaaggaac	
*Gata4 P5*	tctgagaggagccgataacc	gaactaggcgacctctgtgc	
*Gata6 P1*	catttggagggagcgactaa	tccaaggacgctagtttggt	
*Gata6 P3*	agaacctggactgcgcttt	tttgctgctccctcaatgta	
*Gata6 P5*	cctggtgtcccaacacacta	tggccttgaattcactccat	
*Gapdh pmt*	gggttcctataaatacggactgc	ctggcactgcacaagaagat	
*Gata4*	ggaagacaccccaatctcg	catggccccacaattgac	qRT-PCR
*Gata6*	ggtctctacagcaagatgaatgg	tggcacaggacagtccaag	
*Dab2*	ccacctccacaaagtaccaaa	caagcaagtcgtttgctgaa	
*Hdac3*	ctctggtgaagggtttggaa	tgtccatgtctcatccctga	
*Gapdh*	agcttgtcatcaacgggaag	tttgatgttagtggggtctcg	Reference gene
*β-Actin*	ctctcttccagccatctttcat	tataggtggtttcgtggatgc	
*Tbp*	gggagaatcatggaccagaa	gatgggaattccaggagtca	
*Ppib*	tcttcataaccacagtcaagacc	accttccgtaccacatccat	
*Hprt*	cctcctcagaccgctttt	cctggttcatcatcgctaatc	
*Ppar*	ccttccctgtgaactgacg	ccacagagcgctaagctgt

**Table 2:** **Stability order of house-keeping genes in NPD and Emb-LPD EBs selected by GeNorm and Normfinder software**

	**GeNorm**	**Normfinder**
Embryoid Body	*Gapdh-Ppib*	*Tbp*
	*Tbp*	*Ppib*
	*Hprt*	*Gapdh*
	*Ppar*	*Hprt*
	*Actin*	*Ppar*
		*Actin*

### Protein isolation and western blotting

Approximately 100 EBs on day 5.5 were washed with PBS and lysed with 120 μl RIPA buffer (50 mM Tris–HCl [pH 7.5], 150 mM NaCl, 1 mM EDTA, 1 mM EGTA, 1% NP-40, 1% sodium deoxycholate, 1 mM Na_3_VO_4_, 50 mM NaF, cOmplete EDTA-free protease inhibitor cocktail (Roche), 0.5 mM PMSF). Lysate was sonicated on ice and protein content detected using the BCA assay (Pierce). 20 μl protein was mixed with 4x LDS sample buffer and DTT and boiled for 5 min before electrophoresis. After electrophoresis, protein was transferred to polyvinylidene fluoride membrane (0.45 μm) using wet transfer protocol. This was followed by blocking with 5% milk in TBS and incubation with primary antibody overnight at 4°C. The next day the membrane was washed with TBST and incubated with IRDye secondary antibody (Odyssey; 1:10,000) for 1 hour, washed and imaged with the Licor western detection system. Band intensity was quantified with Licor software. Antibodies used are shown in Table 3. α-tubulin was used as loading control and did not change in expression with respect to dietary treatment (data not shown).

**Table 3:** **Antibodies used in western blotting and ChIP analyses**

**Antibody**	**Source**	**Code/dilution**	**Technique**
Gata6	R&D Systems	AF1700, 1:100	westerns
Dab-2	BD Biosciences	610465, 1:1,000	westerns
α-tubulin	Cell Signaling	2144, 1:500	westerns
H4Ac	Millipore	06-866	ChIP
H3Ac	Millipore	06-599	ChIP
H3K4Me3	Actif Motif	339916	ChIP
H3K4Me2	Actif Motif	339914	ChIP
H3K9Me3	Abcam	ab8898	ChIP
H3K27Me3	Abcam	ab6002	ChIP
RNA Polymerase II	Abcam	ab5408	ChIP

### Chromatin immunoprecipitation (ChIP)

A modified protocol was used to perform the Matrix ChIP assay [42]. EBs were dissociated with trypsin EDTA for 5 mins at 37°C, then serum was added to stop trypsinisation and the sample passed through a 21 g needle to form a single cell suspension, diluted to 1×10^6^ per ml, cross-linked with 0.4% formaldehyde for 15 min at RT, then quenched with 125 mM glycine. Cells were spun down and resuspended in IP buffer (150 mM NaCl, 50 mM Tris–HCl pH 7.5, 5 mM EDTA pH 8, 0.5% NP-40, 0.5% Triton-X-100) supplemented with proteinase and phosphatase inhibitors (cOmplete EDTA-free protease inhibitor cocktail, Roche; Phosphatase Inhibitor Cocktail 2 and 3, Sigma) added to suspension (200 μl per 1x10^6^ cells) and incubated on ice for 5 mins before centrifugation at 10,000 rpm for 3 mins at 4°C to obtain nuclei-enriched pellet. Pellets were suspended in the same amount of IP buffer with proteinase inhibitor (200 μl per 1x10^6^ cell nuclei). DNA was sheared on ice with MSE Soniprep 150 (6 μA for 20 cycles of 30 s on/ 60s off followed by 7.5 μA for 4 cycles of 10s on/ 30 s off). 96-well polypropylene PCR plates treated with UV-C light for 2 days were used for ChIP. Each well was incubated with 0.5 μg Protein A (Sigma) in 100 μl 1 × PBS for 36 hours. On the day of use, each plate was washed twice with 100 μl 1 × PBS, then blocked with 200 μl blocking buffer (IP buffer with addition of 5% BSA and 100 μg/ml sheared salmon sperm DNA) for 30 mins, RT. Wells were then incubated with 0.25 μg antibody in 50 μl blocking buffer for 2 hours, RT. Chromatin (equivalent of 5x10^4^ cells per assay) was diluted to 50 μl with blocking buffer, pre-incubated for 15 mins on ice and then incubated in the wells for 2.5 hours at 4°C. Wells were washed with 100 μl ice-cold IP buffer 7 times and twice with 100 μl ice cold TE buffer (10 mM Tris, 1 mM EDTA; pH 7.6). Finally, 50 μl elution buffer (0.1 mg/ml proteinase K, 25 mM Tris base, 1 mM EDTA, pH 10) was added per well. 1/10th of the sample chromatin in 50 μl of elution buffer was used as input. Plates were incubated for 30 min at 55°C, followed by 10 min at 95°C in an ABI 7900HT thermocycler prior to quantitative PCR (qPCR).

Antibodies used in ChIP are shown in Table 3. Each qPCR reaction used 2 μl immunoprecipitated DNA. The modified histone binding was expressed as fold enrichment ratio to IgG negative control (Cell Signaling). Standards and samples were simultaneously amplified in 10 μl reaction volume and primers were designed to amplify genomic sequences at the 5′UTR of *Gata4*, *Gata6* and *Gapdh* genes (see Table 1).

### Statistical analysis

Results were expressed as mean ± S.E.M. Comparison of mRNA expression, histone modification or Western blotting between control and treated groups was performed by one way ANOVA or t-test. EB diameters were compared across treatments using multilevel random effects regression model (SPSS). Significance testing was set at P < 0.05 (two-tailed).

## Abbreviations

ChIP: Chromatin immunoprecipitation
DOHaD: Developmental Origins of Health and Disease
Emb-LPD: Embryo low protein diet
EB: Embryoid body
ES: Embryonic stem cells
HDAC: Histone deacetylase
ICM: Inner cell mass
NPD: Normal protein diet
PE: Primitive endoderm
TE: Trophectoderm
VYS: Visceral yolk sac

## References

[1] Fleming TP, Kwong WY, Porter R, Ursell E, Fesenko I, Wilkins A (2004). The embryo and its future. Biol Reprod.

[2] Fleming TP, Velazquez MA, Eckert JJ, Lucas ES, Watkins AJ (2012). Nutrition of females during the peri-conceptional period and effects on foetal programming and health of offspring. Anim Reprod Sci.

[3] Steegers-Theunissen RP, Twigt J, Pestinger V, Sinclair KD (2013). The periconceptional period, reproduction and long-term health of offspring: the importance of one-carbon metabolism. Hum Reprod Update.

[4] Barker DJ (2007). The origins of the developmental origins theory. J Intern Med.

[5] Barker DJ, Thornburg KL (2013). The obstetric origins of health for a lifetime. Clin Obstet Gynecol.

[6] Langley-Evans SC (2013). Fetal programming of CVD and renal disease: animal models and mechanistic considerations. Proc Nutr Soc.

[7] Hanson MA, Gluckman PD (2008). Developmental origins of health and disease: new insights. Basic Clin Pharmacol Toxicol.

[8] Kwong WY, Wild AE, Roberts P, Willis AC, Fleming TP (2000). Maternal undernutrition during the preimplantation period of rat development causes blastocyst abnormalities and programming of postnatal hypertension. Development.

[9] Watkins AJ, Ursell E, Panton R, Papenbrock T, Hollis L, Cunningham C (2008). Adaptive responses by mouse early embryos to maternal diet protect fetal growth but predispose to adult onset disease. Biol Reprod.

[10] Eckert JJ, Porter R, Watkins AJ, Burt E, Brooks S, Leese HJ (2012). Metabolic induction and early responses of mouse blastocyst developmental programming following maternal low protein diet affecting life-long health. PLoS One.

[11] Sun C, Velazquez MA, Marfy-Smith S, Sheth B, Cox A, Johnston DA (2014). Mouse early extra-embryonic lineages activate compensatory endocytosis in response to poor maternal nutrition. Development.

[12] Coan PM, Vaughan OR, McCarthy J, Mactier C, Burton GJ, Constancia M (2011). Dietary composition programmes placental phenotype in mice. J Physiol.

[13] Young LE, Fernandes K, McEvoy TG, Butterwith SC, Gutierrez CG, Carolan C (2001). Epigenetic change in IGF2R is associated with fetal overgrowth after sheep embryo culture. Nat Genet.

[14] Fernandez-Gonzalez R, Moreira P, Bilbao A, Jimenez A, Perez-Crespo M, Ramirez MA (2004). Long-term effect of in vitro culture of mouse embryos with serum on mRNA expression of imprinting genes, development, and behavior. Proc Natl Acad Sci U S A.

[15] Mann MR, Lee SS, Doherty AS, Verona RI, Nolen LD, Schultz RM (2004). Selective loss of imprinting in the placenta following preimplantation development in culture. Development.

[16] Morgan HD, Jin XL, Li A, Whitelaw E, O'Neill C (2008). The culture of zygotes to the blastocyst stage changes the postnatal expression of an epigentically labile allele, agouti viable yellow, in mice. Biol Reprod.

[17] Rivera RM, Stein P, Weaver JR, Mager J, Schultz RM, Bartolomei MS (2008). Manipulations of mouse embryos prior to implantation result in aberrant expression of imprinted genes on day 9.5 of development. Hum Mol Genet.

[18] Rossant J, Chazaud C, Yamanaka Y (2003). Lineage allocation and asymmetries in the early mouse embryo. Philos Trans R Soc Lond B Biol Sci.

[19] Schrode N, Saiz N, Di Talia S, Hadjantonakis AK (2014). GATA6 levels modulate primitive endoderm cell fate choice and timing in the mouse blastocyst. Dev Cell.

[20] Artus J, Piliszek A, Hadjantonakis AK (2011). The primitive endoderm lineage of the mouse blastocyst: sequential transcription factor activation and regulation of differentiation by Sox17. Dev Biol.

[21] Morrisey EE, Musco S, Chen MY, Lu MM, Leiden JM, Parmacek MS (2000). The gene encoding the mitogen-responsive phosphoprotein Dab2 is differentially regulated by GATA-6 and GATA-4 in the visceral endoderm. J Biol Chem.

[22] Sun-Wada GH, Manabe S, Yoshimizu T, Yamaguchi C, Oka T, Wada Y (2000). Upstream regions directing heart-specific expression of the GATA6 gene during mouse early development. J Biochem.

[23] Caslini C, Capo-chichi CD, Roland IH, Nicolas E, Yeung AT, Xu XX (2006). Histone modifications silence the GATA transcription factor genes in ovarian cancer. Oncogene.

[24] Bartova E, Krejci J, Harnicarova A, Galiova G, Kozubek S (2008). Histone modifications and nuclear architecture: a review. J Histochem Cytochem.

[25] Liu X, Zhao D, Zheng Y, Wang L, Qian Y, Xu C (2014). Expression of histone acetyltransferase GCN5 and histone deacetylase 1 in the cultured mouse preimplantation embryos. Curr Pharm Des.

[26] Leahy A, Xiong JW, Kuhnert F, Stuhlmann H (1999). Use of developmental marker genes to define temporal and spatial patterns of differentiation during embryoid body formation. J Exp Zool.

[27] Doughton G, Wei J, Tapon N, Welham MJ, Chalmers AD (2014). Formation of a polarised primitive endoderm layer in embryoid bodies requires fgfr/erk signalling. PLoS One.

[28] Chlon TM, Crispino JD (2012). Combinatorial regulation of tissue specification by GATA and FOG factors. Development.

[29] Molkentin JD (2000). The zinc finger-containing transcription factors GATA-4, −5, and −6. Ubiquitously expressed regulators of tissue-specific gene expression. J Biol Chem.

[30] Capo-chichi CD, Roland IH, Vanderveer L, Bao R, Yamagata T, Hirai H (2003). Anomalous expression of epithelial differentiation-determining GATA factors in ovarian tumorigenesis. Cancer Res.

[31] Cai KQ, Caslini C, Capo-chichi CD, Slater C, Smith ER, Wu H (2009). Loss of GATA4 and GATA6 expression specifies ovarian cancer histological subtypes and precedes neoplastic transformation of ovarian surface epithelia. PLoS One.

[32] Morrisey EE, Tang Z, Sigrist K, Lu MM, Jiang F, Ip HS (1998). GATA6 regulates HNF4 and is required for differentiation of visceral endoderm in the mouse embryo. Genes Dev.

[33] Capo-Chichi CD, Smedberg JL, Rula M, Nicolas E, Yeung AT, Adamo RF (2010). Alteration of Differentiation Potentials by Modulating GATA Transcription Factors in Murine Embryonic Stem Cells. Stem Cells Int.

[34] Padua MB, Fox SC, Jiang T, Morse DA, Tevosian SG (2014). Simultaneous gene deletion of gata4 and gata6 leads to early disruption of follicular development and germ cell loss in the murine ovary. Biol Reprod.

[35] Carrasco M, Delgado I, Soria B, Martin F, Rojas A (2012). GATA4 and GATA6 control mouse pancreas organogenesis. J Clin Invest.

[36] Zhao R, Watt AJ, Battle MA, Li J, Bondow BJ, Duncan SA (2008). Loss of both GATA4 and GATA6 blocks cardiac myocyte differentiation and results in acardia in mice. Dev Biol.

[37] Yang DH, Cai KQ, Roland IH, Smith ER, Xu XX (2007). Disabled-2 is an epithelial surface positioning gene. J Biol Chem.

[38] Capo-Chichi CD, Rula ME, Smedberg JL, Vanderveer L, Parmacek MS, Morrisey EE (2005). Perception of differentiation cues by GATA factors in primitive endoderm lineage determination of mouse embryonic stem cells. Dev Biol.

[39] Watkins AJ, Platt D, Papenbrock T, Wilkins A, Eckert JJ, Kwong WY (2007). Mouse embryo culture induces changes in postnatal phenotype including raised systolic blood pressure. Proc Natl Acad Sci U S A.

[40] Yasuda E, Seki Y, Higuchi T, Nakashima F, Noda T, Kurosawa H (2009). Development of cystic embryoid bodies with visceral yolk-sac-like structures from mouse embryonic stem cells using low-adherence 96-well plate. J Biosci Bioeng.

[41] Lucas ES, Watkins AJ, Cox AL, Marfy-Smith SJ, Smyth N, Fleming TP (2011). Tissue-specific selection of reference genes is required for expression studies in the mouse model of maternal protein undernutrition. Theriogenology.

[42] Flanagin S, Nelson JD, Castner DG, Denisenko O, Bomsztyk K (2008). Microplate-based chromatin immunoprecipitation method, Matrix ChIP: a platform to study signaling of complex genomic events. Nucleic Acids Res.

